# Mobile Technology for Vegetable Consumption: A Randomized Controlled Pilot Study in Overweight Adults

**DOI:** 10.2196/mhealth.5146

**Published:** 2016-05-18

**Authors:** Sarah Ann Mummah, Maya Mathur, Abby C King, Christopher D Gardner, Stephen Sutton

**Affiliations:** ^1^ Stanford Prevention Research Center Stanford University School of Medicine Stanford, CA United States; ^2^ Cambridge Institute of Public Health University of Cambridge School of Clinical Medicine Cambridge United Kingdom; ^3^ Quantitative Sciences Unit Stanford University School of Medicine Stanford, CA United States

**Keywords:** health behavior, cell phones, telemedicine, eating, diet, vegetables, randomized controlled trial, pilot projects

## Abstract

**Background:**

Mobile apps present a potentially cost-effective tool for delivering behavior change interventions at scale, but no known studies have tested the efficacy of apps as a tool to specifically increase vegetable consumption among overweight adults.

**Objective:**

The purpose of this pilot study was to assess the initial efficacy and user acceptability of a theory-driven mobile app to increase vegetable consumption.

**Methods:**

A total of 17 overweight adults aged 42.0 (SD 7.3) years with a body mass index (BMI) of 32.0 (SD 3.5) kg/m^2^ were randomized to the use of Vegethon (a fully automated theory-driven mobile app enabling self-monitoring of vegetable consumption, goal setting, feedback, and social comparison) or a wait-listed control condition. All participants were recruited from an ongoing 12-month weight loss trial (parent trial). Researchers who performed data analysis were blinded to condition assignment. The primary outcome measure was daily vegetable consumption, assessed using an adapted version of the validated Harvard Food Frequency Questionnaire administered at baseline and 12 weeks after randomization. An analysis of covariance was used to assess differences in 12-week vegetable consumption between intervention and control conditions, controlling for baseline. App usability and satisfaction were measured via a 21-item post-intervention questionnaire.

**Results:**

Using intention-to-treat analyses, all enrolled participants (intervention: 8; control: 9) were analyzed. Of the 8 participants randomized to the intervention, 5 downloaded the app and logged their vegetable consumption a mean of 0.7 (SD 0.9) times per day, 2 downloaded the app but did not use it, and 1 never downloaded it. Consumption of vegetables was significantly greater among the intervention versus control condition at the end of the 12-week pilot study (adjusted mean difference: 7.4 servings; 95% CI 1.4-13.5; *P*=.02). Among secondary outcomes defined a priori, there was significantly greater consumption of green leafy vegetables, cruciferous vegetables, and dark yellow vegetables (adjusted mean difference: 2.6, 1.6, and 0.8 servings; 95% CI 0.1-5.0, 0.1-3.2, and 0.3-1.4; *P*=.04, *P*=.04, and *P*=.004, respectively). Participants reported positive experiences with the app, including strong agreement with the statements “I have found Vegethon easy to use” and “I would recommend Vegethon to a friend” (mean 4.6 (SD 0.6) and 4.2 (SD 0.8), respectively, (on a 5-point scale).

**Conclusions:**

Vegethon demonstrated initial efficacy and user acceptability. A mobile app intervention may be useful for increasing vegetable consumption among overweight adults. The small sample size prevented precise estimates of effect sizes. Given the improved health outcomes associated with increases in vegetable consumption, these findings indicate the need for larger, longer-term evaluations of Vegethon and similar technologies among overweight adults and other suitable target groups.

**Trial Registration:**

ClinicalTrials.gov NCT01826591; https://clinicaltrials.gov/ct2/show/NCT01826591 (Archived by WebCite at http://www.webcitation.org/6hYDw2AOB)

## Introduction

Inadequate consumption of vegetables and fruits is responsible for up to 2.6 million deaths worldwide, according to a 2003 estimate by the World Health Organization [1]. Greater consumption of vegetables and fruits is associated with reduced risks of cardiovascular disease, stroke, cancer, and all-cause mortality [1-8]. It has been suggested that these protective effects are greater for vegetables than for fruits [8] and follow a dose-response relationship [6,8,9] with benefits seen in up to 7+ servings daily [8]. Vegetables in particular are rich in phytochemicals, including vitamins and trace minerals, that may protect cells against carcinogenesis [10,11]. They are also high in water and fiber and can promote weight loss and weight management by reducing energy density, promoting satiety, and decreasing energy intake [12,13]. In recognition of these many benefits, a national “5-a-day” campaign was launched in the United States in 1991 to encourage greater consumption of vegetables and fruits. However, Americans' consumption of vegetables significantly decreased during the subsequent decade [14], and despite current United States Department of Agriculture (USDA) recommendations to consume 5-6 servings of vegetables per day [12], US adults consume an average of just 1.7 (standard error (SE) 0.03) servings of vegetables (excluding fried potatoes) each day [14].

Behavioral interventions to increase vegetable and fruit consumption have led to modest increases in daily intake [15]. However, the typical face-to-face approaches used in behavioral interventions to achieve and sustain increased vegetable consumption often cannot be realistically implemented at the population level [15], particularly given the high cost of most current strategies [16] (eg, time demands, the need for trained staff, and so forth [17]). Mobile apps present an attractive alternative for delivering scalable dietary behavior change interventions for a number of reasons [18]. Rates of mobile phone adoption among US adults have increased dramatically during recent years, from 35% in 2011 to 56% in 2013 [19], making mobile phone-based interventions ripe for dissemination to whole populations. Individuals' tendencies to carry their phones with them everywhere means that such interventions can provide significantly more touch points, reaching individuals at nearly any time or place [20]. Additionally, the rapidly improving technical capabilities of mobile phones enable the potential for timely feedback, personalization, and interactivity to maximize the potential effectiveness of interventions over time [21].

An explosion of health-promoting mobile apps has occurred in recent years [22] including apps to promote weight control [23], healthier eating [24-26], and greater vegetable consumption [27]. However, most mobile health (mHealth) apps are yet to undergo evaluation in randomized trials or incorporate theory-based strategies known to drive changes in health behaviors [28-30]. Theory-driven behavior change techniques [31] may form the basis of apps that more effectively produce improvements in health behaviors. Investigators have called for both the theory-informed development and rigorous evaluation of mobile phone-based interventions [32,33]. With the mHealth field still in its infancy, it is likely that the most effective approaches are yet to be explored.

This pilot study (trial registration: ClinicalTrials.gov NCT01826591) aimed to assess the initial efficacy and user acceptability of Vegethon, a stand-alone mobile app designed to increase vegetable consumption through the creative application of behavior change theory and techniques. It is among the first apps specifically targeting only vegetable consumption to undergo evaluation in a randomized controlled study of adults attempting to lose weight.

## Methods

### Study Design

A randomized controlled study design was used, with a 1:1 allocation ratio of intervention to wait-listed control (12-week delay) participants. During a pre-randomization orientation session taking place face-to-face, participants were instructed that the mobile app was intended to support them in increasing their vegetable consumption and that it was ideally to be used for 1-2 minutes on a daily basis. They were instructed to avoid using any other apps focused on vegetable consumption for the duration of the study. Participants providing written consent were requested to complete a Web-based, self-administered baseline questionnaire. All participants who completed the questionnaire were randomized. Participants in the intervention condition were instructed to use the app for a minimum of 6 weeks. Outcome data were collected from both conditions using a Web-based questionnaire self-administered 12 weeks after randomization. To prevent contamination among conditions, the app was made available to intervention participants only through the distribution of single-use registration codes. The CONSORT-EHEALTH guidelines were followed in reporting this study (Multimedia Appendix 1).

### Participants

Participants were overweight adults motivated to lose weight and eat healthier. Individuals were recruited from an ongoing 12-month weight loss trial (n=609) based at Stanford University (parent trial), in which participants were aged 18-50 years, had an initial body mass index (BMI) of 28-40 kg/m^2^, were non-diabetic and non-hypertensive, had no cancer or heart, renal, or liver disease, and lived in the geographical area surrounding Stanford. The added eligibility criterion for this pilot study was ownership of an iPhone. All participants provided written informed consent, and this research was approved by the Stanford University Human Subjects Committee.

### Parent Trial

The study was implemented during months 7-10 (ie, maintenance phase) of the parent trial. In the parent trial, participants were randomized to either a low-fat or a low-carbohydrate diet for 12 months and attended 22 evening classes led by a health educator. Both parent trial treatment groups were encouraged to include vegetables in their daily diets. Parent trial participants who chose to concurrently participate in this pilot study were re-randomized to receipt of the mobile app or a wait-listed control condition. Thus, any potential spillover effects of the parent trial on vegetable consumption affected both intervention and control conditions in this study.

### Randomization

Participants were randomized and assigned prospectively via a balanced assignment approach designed to ensure balance across mobile intervention assignment and both elements of parent trial treatment assignment, consisting of (1) diet group (low-carbohydrate or low-fat) and (2) health educator (of 4 possible health educators). Because the randomized parent trial was underway at the time of this study, the diet group and health educator could not be manipulated and were instead treated as nested strata. Participants in this study were randomly assigned within these strata via a randomized, balanced block size of 4; subsequent participant assignments were selected via an a priori, deterministic procedure. This randomization assignment procedure was preferable to other randomization approaches because it ensured balance in all 3 variables at any given sample size of this pilot trial. Additionally, it was preferable over sequentially adjusted randomization procedures such as Efron's biased coin [34] because it allowed all assignments to be created prospectively.

### Intervention

Participants randomized to the pilot study intervention condition completed a short Web-based tutorial that described the fully automated mobile app and its use. The tutorial guided them through the process of downloading the app onto their iPhones from the iTunes App Store on December 4, 2014, creating a user account with their individual registration code, and setting their initial goals for quantity and variety of vegetable consumption.

Vegethon [35] was a stand-alone mobile app that enabled self-monitoring of vegetable consumption and included a constellation of theory-driven features to maximize behavior change and sustain user engagement. Formative research indicated that this target population desired a simple and efficient means of self-monitoring. Self-monitoring is among the most widely used behavior change techniques in mobile apps to change health behavior [36]. It is acknowledged to be a critical component of behavior change interventions [37] and can be viewed as part of the process of self-regulating behavior [38]. Vegethon enabled swift vegetable logging by tapping on different vegetable icons (eg, eggplant, arugula) to indicate the number of servings of each vegetable consumed. To facilitate weight loss and weight maintenance in the context of the parent trial, the app focused on non-starchy vegetables with lower energy density and excluded starchy vegetables such as potatoes and corn. Tapping on each vegetable icon increased its quantity in increments of 1/2 servings. To reduce the cognitive load associated with self-monitoring, users were instructed by the app to estimate 1 vegetable serving as approximately the size of their fist. Users were able to select and modify goals for the quantity and variety of their daily vegetable consumption on 2 sliding scales ranging from 1 to 10 servings or types, each anchored by an “average” value of 2, “recommended” value of 5, and “superstar” value of 8. Default values [39] were set to the recommended 5 servings and 5 types of vegetables.

Overall, the intervention was grounded in behavioral theory, emphasizing the importance of centering an intervention around the *process* of behavior change (eg, the fun of tapping on colorful vegetable icons; the pride in surpassing a friend's vegetable score). This process motivation strategy stands in contrast to interventions focusing on the eventual outcomes of behavior change (eg, improved health) that can often be too far in the future to motivate and sustain behavior change [40]. Several theory-driven elements aiming to increase this type of process motivation complemented the primary self-monitoring component of Vegethon. To increase elements of fun and challenge, for example, 7 ongoing challenges were included that ranged from easy to difficult to perform (eg, *Breakfast Champ: Eat any vegetable before 11 am*). Similarly, elements of surprise and choice were incorporated through surprise challenges delivered to users via push notifications every 4 days, enabling the selection of a desired challenge. Competition, which has been shown by Lepper and colleagues [41] to increase intrinsic motivation for behavior change, was applied through a leaderboard in which users competed against “other Vegethoners” who were “most similar” to them. Finally, to foster a process of identity revision toward one who is a vegetable eater [42], each participant was referred to as a *Vegethoner* throughout the intervention.

As with most mHealth interventions, the intervention made use of opportunities for just-in-time feedback in a number of ways. Short-term progress monitoring was displayed with vertical bar graphs showing the current day's goals versus consumption. Long-term progress monitoring was displayed with horizontal bar graphs showing consumption for each of the previous 7 days and 7 weeks. Feedback on the fulfillment of goals and challenges was reinforced through in-app notifications. Just-in-time prompts to log vegetables were delivered most evenings at 9 pm through push notifications.

### Blinding

Researchers who performed data analysis were blinded to condition assignment. Randomization was performed by a researcher who had no contact with participants and used a random, computer-generated allocation sequence to assign participants to each condition. Enrollment and post-randomization communication with participants were subsequently performed by a research assistant who did not play a role in the data analysis. All parent trial staff members, including the dietitians leading the health education classes, were blinded to condition assignment, and participants were instructed not to discuss the app with their health educator or with other participants.

### Data collection

#### App Usage

App usage was measured using inbuilt software tracking the date of app download, number of servings logged for each vegetable type, and time and date that logging occurred. Data were tracked for each participant using their individually assigned registration codes.

#### Vegetable Consumption

To assess the primary outcome of daily vegetable consumption at baseline and 12 weeks after randomization, an adapted version of the validated semiquantitative Harvard Food Frequency Questionnaire (FFQ) was used [43,44]. As with the Harvard FFQ, commonly used portion sizes for each vegetable (eg, 1 onion, 1/2 cup of broccoli) were specified, and participants were asked to indicate how often, on average, during the past week, they had consumed each type and amount of food. All 28 questions on vegetable consumption comprising 33 vegetables were included from the Harvard FFQ, and 8 response categories were possible, ranging from 0 to ≥6 times per day [45]. Daily vegetable intake was calculated for each participant following an established method [46,47] in which the daily frequency of consumption for each vegetable item was multiplied by the number of servings represented by the specified portion size, based on USDA guidelines [48], and subsequently combined to yield total daily vegetable servings. Vegetable subgroups were defined a priori based on established criteria [49] adapted for specific use with the Harvard FFQ [50,51]. Subgroups included green leafy vegetables (kale, mustard greens, chard, spinach, iceberg or head lettuce, romaine or leaf lettuce), cruciferous vegetables (broccoli, cauliflower, cabbage, coleslaw, brussels sprouts), dark yellow vegetables (carrots, carrot juice, yams, sweet potatoes, dark orange winter squash), tomatoes (tomatoes, tomato juice, V8 juice, tomato sauce, salsa, picante or taco sauce), beans/lentils (beans, lentils, tofu, soy burger or other soy protein), and other vegetables (eggplant, zucchini or other summer squash, celery, string beans, peas, lima beans, corn, mixed vegetables, stir-fry, vegetable soup, green or red peppers, onions). All vegetable subgroups apart from beans/lentils were assessed as secondary outcomes. Beans/lentils, which were not targeted by the intervention, were assessed as a control measure.

#### App Usability and Satisfaction

Usability of and satisfaction with the mobile app were assessed using a 21-item questionnaire, administered 12 weeks after randomization to the intervention condition only, and adapted from similar surveys used by King et al [52] to assess user acceptability of mHealth interventions. Participants were asked to rate their level of agreement or disagreement with each statement on a 5-point Likert-type scale.

### Statistical Analysis

A convenience sample drawn from the parent trial was used for this pilot study. An analysis of covariance (ANCOVA) was used to assess differences between intervention and control conditions, with vegetable consumption as the dependent variable, condition assignment as the fixed factor, and baseline value of the dependent variable as a covariate. The use of ANCOVA was appropriate for a small sample size as it can be considered a special case of regression, in which a conservative rule of thumb is to have at least 10 subjects per predictor variable; in the present analysis with one predictor variable, a minimum total sample size of 10 was reasonable to satisfy parametric assumptions and minimize the chance of overfitting [53]. An intention-to-treat analysis was used such that baseline observations were carried forward when participants were lost to follow-up. A sensitivity analysis with multiple imputations to account for missing data was also used. Five imputed datasets were created using the chained equations method and pooled estimates using Barnard-Rubin adjusted degrees of freedom [54,55]. Descriptive statistics were used to analyze participant baseline characteristics and user acceptability of the mobile app. SPSS Statistics software version 22.0 (IBM, New York) was used.

## Results

### Participants

**Figure 1:**
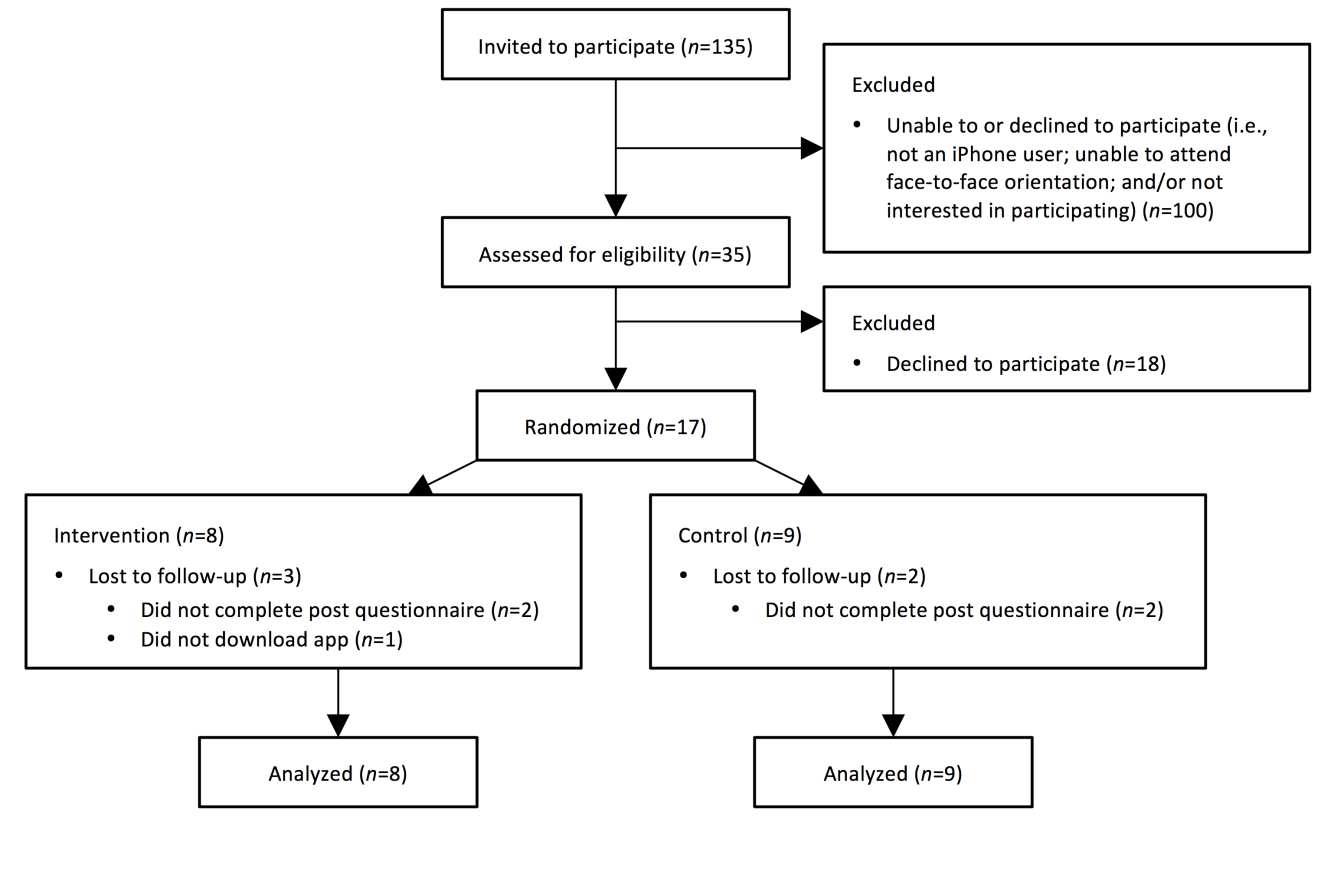
CONSORT flowchart. An intention-to-treat analysis was used.

Participant study flow is presented in Figure 1. Enrollment began in October 2014, and the study ended in February 2015. A subset of participants (n=135) enrolled in the parent trial at a time point coinciding with this pilot study were invited to participate. Thirty-five participants responded to an email indicating that they (1) had an iPhone, (2) wished to participate in a mobile app substudy, and (3) were willing to attend a face-to-face orientation session. Among these 35 individuals, 17 completed a baseline questionnaire and were randomized and analyzed (intervention: 8; control: 9). Participant baseline characteristics are summarized in Table 1. Although there were more women than men in the control versus intervention conditions, the two conditions were comparable in age, BMI, and race and ethnicity. Randomization resulted in 2 conditions that were balanced across parent trial treatment assignment and parent trial health educator (Table 1).

**Table 1:** Participant baseline characteristics.

Characteristic		Control	Intervention
Gender, n (%)			
	Female	7 (78)	4 (50)
	Male	2 (22)	4 (50)
Age in years, mean (SD)		41.2 (7.6)	42.9 (7.3)
BMI^a^ in kg/m^2^, mean (SD)		31.7 (3.8)	32.3 (3.3)
Race/ethnicity, n			
	White	7	6
	Asian	1	0
	Other	1	2
Parent trial treatment assignment, n			
	Low-carbohydrate diet	5	4
	Low-fat diet	4	4
Parent trial health educator assignment, n			
	A	3	2
	B	3	3
	C	1	2
	D	2	1

^a^BMI: body mass index.

### Retention and Adherence

Among the 17 enrolled participants, 75% (6/8) of the intervention condition and 78% (7/9) of the control condition completed the post questionnaire administered 12 weeks after randomization. As noted earlier, an intention-to-treat approach was applied in analyzing the study data. Of the 8 participants who were randomized to the intervention, 5 downloaded the app within 2 days, 2 downloaded it within 13 days but did not use it, and 1 never downloaded it.

### App Usage

**Figure 2:**
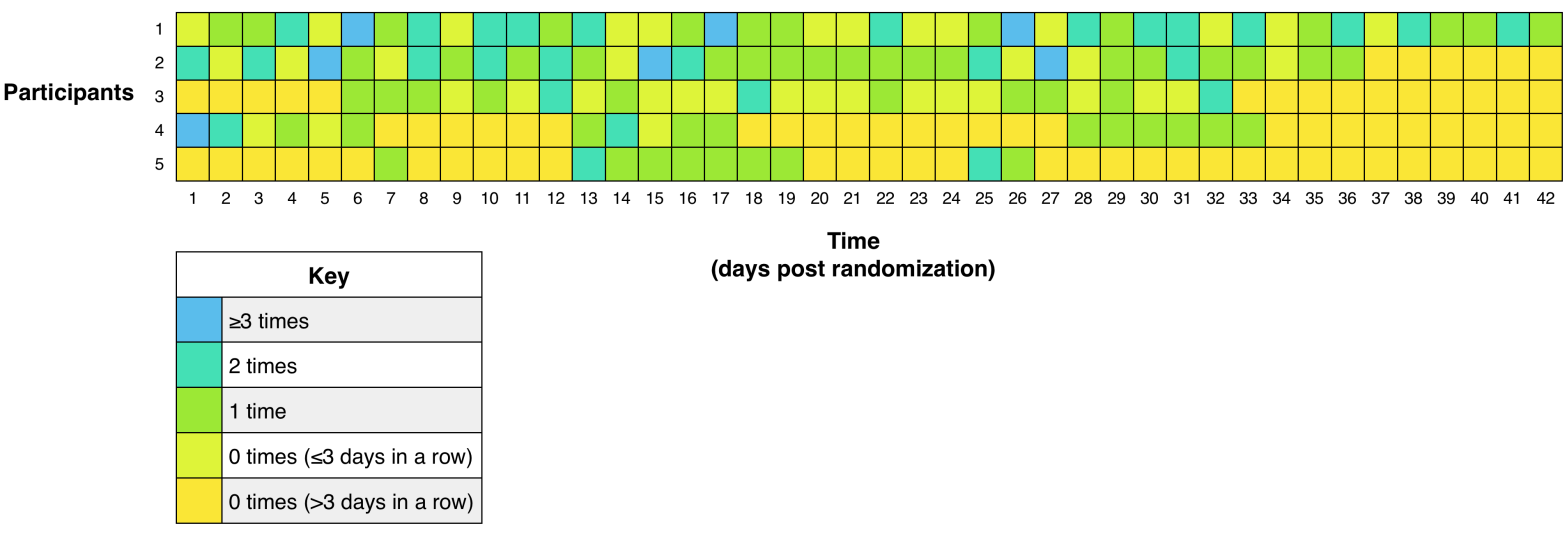
Frequency of vegetable logging among intervention condition, during 6-week intervention period. Day 22 was Christmas Day (Dec 25).

Among those in the intervention condition who used the app (5 of 8 participants), participants logged their vegetable consumption a mean of 0.7 (SD 0.9) times per day. Daily frequency of vegetable logging over the course of the 6-week intervention period is presented in Figure 2. There was a downward trend in frequency of logging behavior over time, from 0.8 (SD 1.2) times per day during week one to 0.3 (SD 0.6) times per day during week six. There was wide variation in logging frequency among individuals, ranging from 1.2 (SD 1.1) to 0.3 (SD 0.6) times per day.

**Figure 3:**
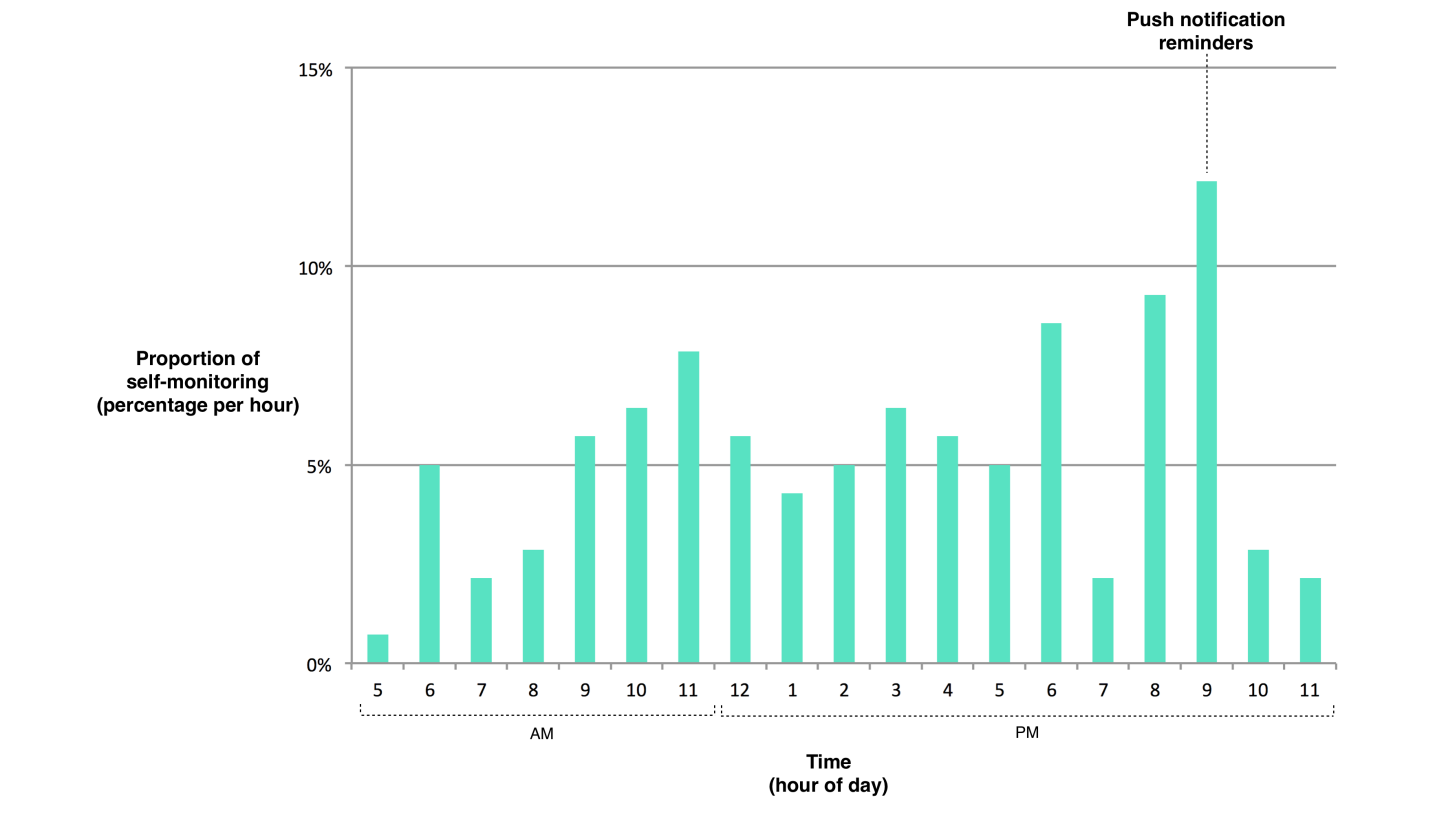
Time of day that users recorded their vegetable consumption using Vegethon. Each hour represents the subsequent 1-hour interval (eg, 5 represents 5:00-5:59 AM). Push notifications were sent at 9 PM as reminders to those who logged the day before but had not yet logged that day.

Vegetable logging occurred during all waking hours, with the greatest proportion of logging taking place during the 1-hour interval after 9 pm (Figure 3). Logging also occurred during all days of the week, with the greatest proportion occurring on Thursdays and the smallest proportion on Wednesdays and Fridays. In examining user logging behavior (ie, the selection of a number of servings consumed per individual vegetable type, such as cucumber), 40% of the time users selected 0.5 servings and 38% of the time users selected 1.0 serving, while all larger serving increments (1.5, 2.0, and so on) were each used less than 9% of the time.

### Vegetable Consumption

**Figure 4:**
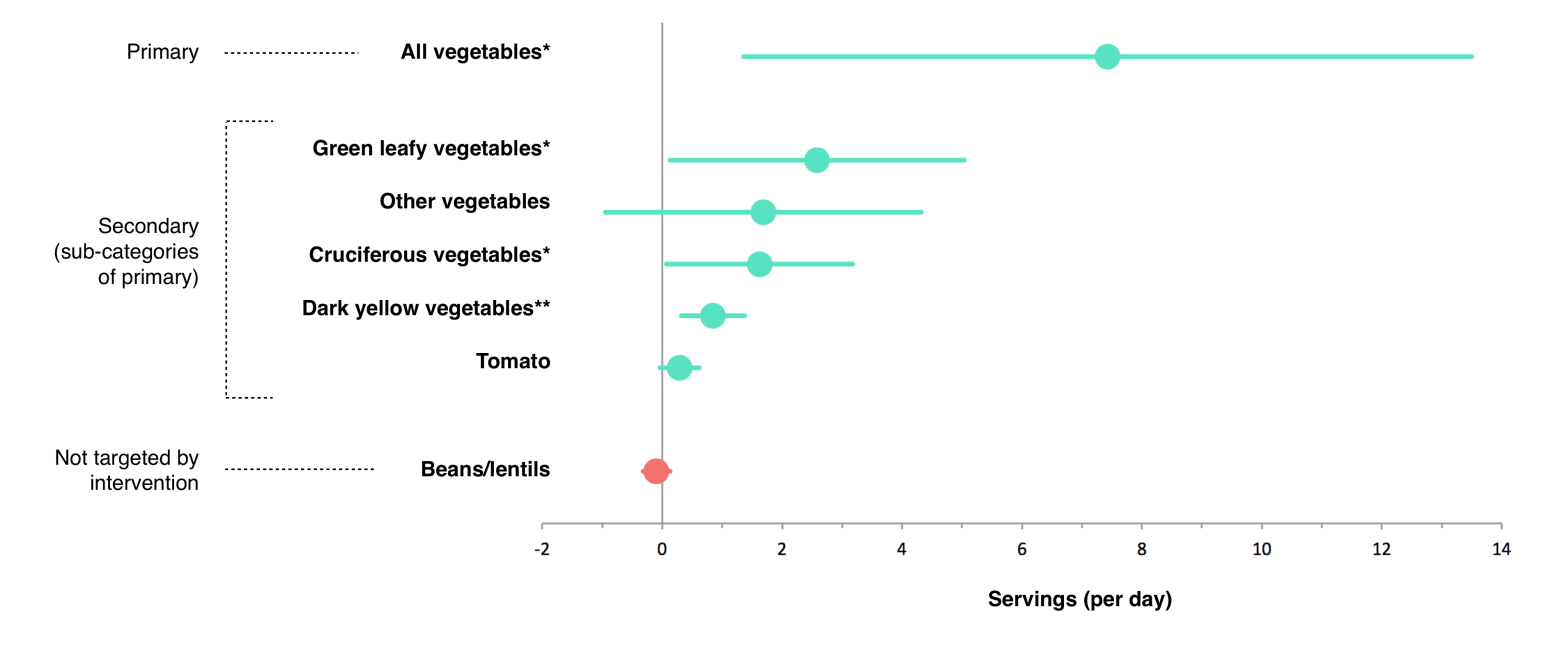
Differences in vegetable consumption, 12 weeks after randomization (n=17). Adjusted mean difference (circles) and 95% confidence intervals (horizontal lines) between intervention condition (mobile app) and control condition (no mobile app): all vegetables 7.4 (1.4-13.5); green leafy vegetable 2.6 (0.1-5.0); other vegetables 1.7 (−0.9 to 4.3); cruciferous vegetables 1.6 (0.7-3.2); dark yellow vegetables 0.8 (0.3-1.4); tomato 0.3 (−0.04 to 0.6); and beans/lentils −0.1 (−0.3 to 0.1). An intention-to-treat analysis was used, with baseline values carried forward when participants were lost to follow-up. Vegetable consumption was self-reported using an adapted version of the validated semiquantitative Harvard Food Frequency Questionnaire. *P<.05 and **P<.01, based on analysis of covariance predicting post-intervention values, controlling for baseline values.

Using intention-to-treat analyses, daily vegetable consumption (primary outcome) was found to be significantly greater in the mobile app intervention compared with the control condition at the end of the 12-week pilot study (adjusted mean difference: 7.4 servings; 95% CI 1.4-13.5; *P*=.02). A multiple imputation sensitivity analysis was performed and did not significantly alter these results (*P*=.03). Among secondary outcomes defined a priori, there was significantly greater consumption of green leafy vegetables (adjusted mean difference: 2.6 servings; 95% CI 0.1-5.0; *P*=.04), cruciferous vegetables (1.6 servings; 95% CI 0.1-3.2; *P*=.04), and dark yellow vegetables (0.8 servings; 95% CI 0.3-1.4; *P*=.004) in the intervention versus control condition. There were also statistically non-significant trends toward greater consumption of tomatoes (0.3 servings; 95% CI 0.0-0.6; *P*=.08) and other vegetables (1.7 servings; 95% CI −0.9 to 4.3; *P*=.19) in the intervention versus control condition. Consumption of beans/lentils, which was not targeted by the intervention, was not significantly different (−0.1 servings; 95% CI −0.3 to 0.1; *P*=.37; Figure 4) between the conditions.

**Figure 5:**
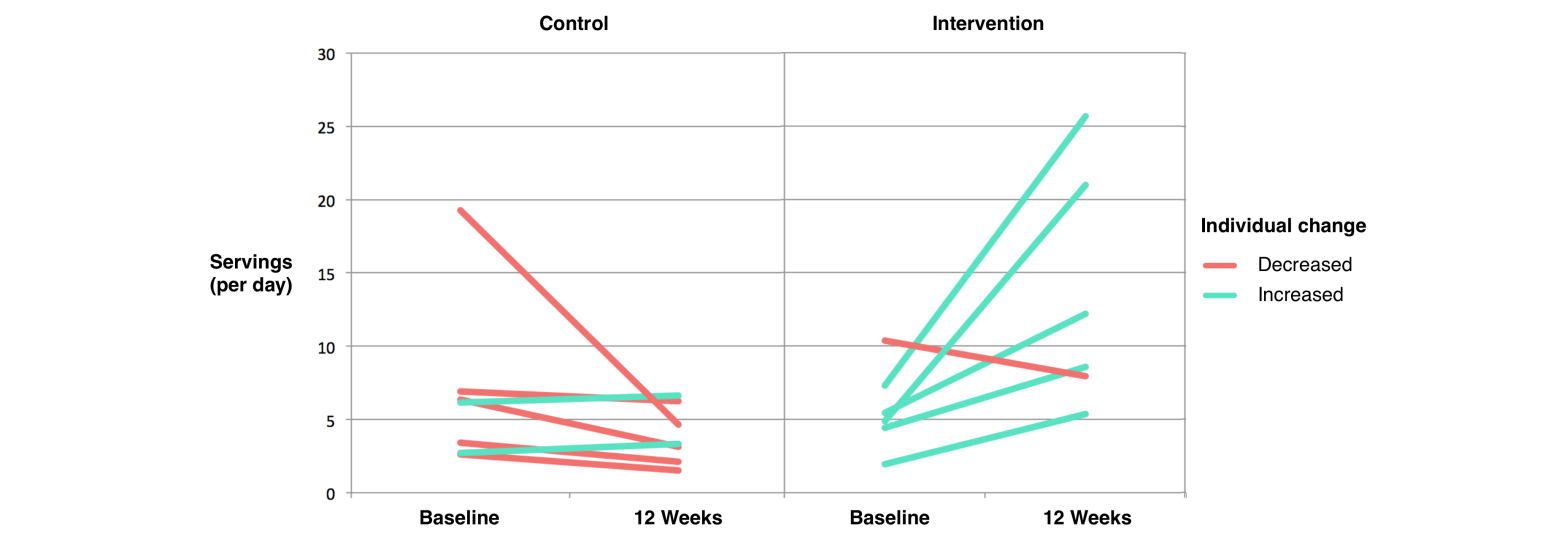
Individual changes in vegetable consumption, from baseline to 12 weeks after randomization (n=13). Vegetable consumption was self-reported using an adapted version of the validated semiquantitative Harvard Food Frequency Questionnaire. This analysis excludes 2 intervention and 2 control condition participants lost to follow-up.

Mean baseline consumption of vegetables was comparable in both conditions. The observed means and SD in the intervention condition were 6.0 (SD 2.7) servings at baseline and 13.5 (SD 8.1) servings at 12 weeks. In the control condition, the observed means were 7.0 (SD 5.9) servings at baseline and 3.9 (SD 2.0) servings at 12 weeks. Individual participant changes in consumption from baseline to 12 weeks are presented in Figure 5. Among intervention participants, vegetable consumption increased in 83% (5/6) of participants completing the FFQ, whereas among control participants, vegetable consumption decreased in 71% (5/7) of participants completing the questionnaire.

### App Usability and Satisfaction

**Figure 6:**
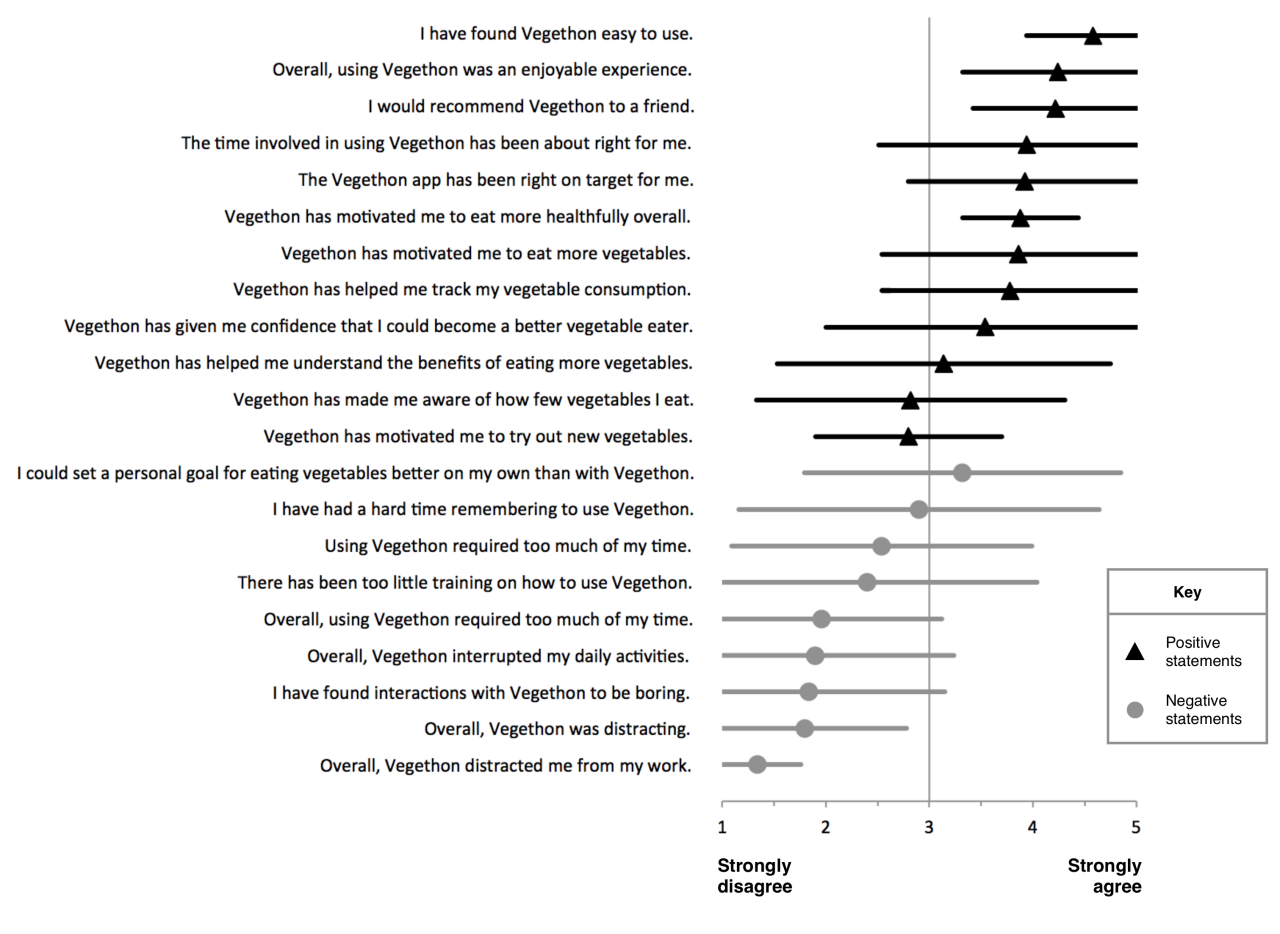
Satisfaction with and usability of mobile app intervention. Mean (triangles and circles) and SD (horizontal lines). This exploratory analysis excludes 2 participants who were lost to follow-up and 1 participant who did not use the app. Participants were asked to rate each statement on a 5-point Likert-type scale.

Intervention participants reported positive experiences with the mobile app, including strongest agreement with the following statements: “I have found Vegethon easy to use”; “Overall, using Vegethon was an enjoyable experience”; and “I would recommend Vegethon to a friend” (mean 4.6 (SD 0.6), 4.2 (SD 0.9), and 4.2 (SD 0.8), respectively, on a 1-5 scale, with 5=strongly agree and 1=strongly disagree; Figure 6). Participants reported strong disagreement with the statements “Overall, Vegethon distracted me from my work”; “I have found interactions with Vegethon to be boring”; and “Overall, using Vegethon required too much of my time” (mean 1.3 (SD 0.4), 1.8 (SD 1.3), and 2.0 (SD 1.2), respectively).

## Discussion

### Principal Findings

This pilot study aimed to determine the initial efficacy and user acceptability of a mobile app intervention designed to increase vegetable consumption through the creative application of behavior change theory and techniques. Twelve-week testing indicated that Vegethon significantly increased consumption of vegetables, including green leafy vegetables, cruciferous vegetables, and dark yellow vegetables, in the small sample being studied. The mobile app intervention achieved reasonably high rates of engagement and was found to be easy and enjoyable to use by the sample of participants. Increases in vegetable logging observed at 9 pm support the use of push notifications for engaging users. High usage of serving increments “0.5” and “1.0” suggests that the availability of half-serving (vs full serving) increments is appropriate for the logging of individual vegetable types. Given the improved health outcomes associated with increases in vegetable consumption, this pilot study suggests the need for larger, longer-term studies of Vegethon and similar technologies among overweight adults and other suitable target groups.

### Comparison with Prior Work

In this first-generation investigation, the mobile app produced relatively large effect sizes, increasing overall vegetable consumption by 7.4 servings. These effects were observed among participants who already had a relatively high baseline vegetable consumption compared with the US national average [14]. The initial effect size observed compares favorably with that of a Web-based intervention in which vegetable and fruit consumption increased by 4.4 servings per day, as assessed by an FFQ [56]. The results also compare favorably with the only other known randomized study of a mobile app targeting vegetable consumption specifically, which was conducted among young adolescent girls [57]. Although the effects are larger than those seen in previous work, it remains difficult to compare effect sizes given the differences in study outcome measures, study populations, and sample sizes. The theory-driven nature of the app [31], including goal-setting [58] and self-monitoring behavior [37] widely acknowledged to be critical components of behavioral mHealth interventions [59], may have led to greater behavioral changes than observed previously. Overall, these findings align with those found in other investigations of mobile apps to change health behaviors, in which acceptability and initial efficacy have been similarly demonstrated [36].

These results warrant further investigation, as increases in vegetable consumption may lead to changes in overall diet composition and weight loss, even in the absence of specific guidance to decrease consumption of other foods [60]. For example, in a study among overweight adults by Norman et al [61], a short message service intervention that increased vegetable consumption led to weight loss. Such interventions that focus on the inherent benefits of the target behavior itself (eg, increasing vegetable consumption) may lead to more sustained behavior changes than those focusing on longer-term goals (eg, weight loss) [40]. Given the high and growing prevalence of overweight in the United States [62], and the fact that people in higher weight categories are more likely to develop chronic diseases associated with excess weight [63], strategies to reduce modifiable risk factors including diet among overweight adults are needed [62,63]. This study demonstrating the initial efficacy of a mobile app to improve diet among highly motivated overweight adults presents one such possible strategy that warrants further investigation.

### Strengths

Among the strengths of this pilot investigation were the theory-based development of the app that helped to ensure it would be engaging to use for the target population as well as the randomized controlled study design that is relatively rare in the mHealth field, in which few technologies are evaluated with rigorous study designs. Pilot randomized controlled trials represent an important phase in the iterative development of effective digital health interventions [64], particularly in the context of the mHealth landscape where developers often skip outcome evaluations altogether, threatening progress in the field [65]. This stand-alone mHealth app was evaluated in the context of an intensive weight loss trial, in which consumption of high-quality whole foods and vegetables was emphasized. The efficacy of the app beyond the effect of the parent trial suggests the potentially high degree of potency of the mobile app intervention, at least during initial use. These effects may be due to the advantages inherent to mobile phone-based interventions, including timely feedback, personalization, and daily interaction [18], as well as the theory-driven nature of the intervention.

### Limitations

There were several methodological limitations to this pilot study. The participants were concurrently enrolled in a weight loss trial and were interested in helping shape the development of mobile technology. Generalizability of these findings to other samples that are not enrolled in a weight loss trial and/or are not as highly interested or motivated to use a mobile app to increase their vegetable consumption is unknown. The loss of 4 participants to follow-up in this short study may have resulted from the high volume of time-intensive tasks (eg, classes, blood draws, questionnaires) simultaneously required of participants by the parent trial. Further studies are indicated to evaluate the efficacy of the app among participants outside of the context of a weight loss intervention who may respond to different types of motivation.

As with all dietary assessment methods, there were inherent limitations to the adapted Harvard FFQ measurement tool used, including reliance on self-report, which could have led to a response bias (eg, participants randomized to the app simply reporting greater vegetable consumption at follow-up versus actually consuming more vegetables). Moreover, the FFQ has been acknowledged to overestimate intakes, particularly for foods consumed rarely and perceived as healthy (eg, vegetables) [66,67]. Despite the high test-retest reliability of the FFQ over time in the control condition and in measures not targeted by the intervention (eg, bean intake), the observed effect size for the primary outcome was notably large. Although the hypothesis was supported, alternative explanations may include that the use of Vegethon caused participants to become more attuned to their vegetable consumption, more aware of the vegetables they consumed in other foods, and/or more accurate or exaggerated reporters of their vegetable consumption.

The small sample size of this study allowed only an imprecise estimate of the effect size for vegetable consumption and prevented analysis of potential mediators and moderators. Studies in other areas have found that the effect sizes of small initial studies in an area often tend to be larger than those found in subsequent larger studies [68]. A larger study is indicated to more precisely estimate differences in vegetable consumption and to assess mediators and moderators of observed changes in dietary behaviors. The 12-week duration of this pilot study provides an assessment only of short-term effects, and a longer trial is warranted to determine whether the observed effects are sustained over a longer period of time.

### Conclusions

The study results suggest that a theory-based mobile app may be a feasible way to increase vegetable consumption among adults attempting to lose weight, at least in the short term. These findings support the need for theory-driven mobile technologies to more effectively produce improvements in health behaviors. Larger-scale and longer trials are necessary to fully evaluate the potential of Vegethon and similar technologies to produce sustained increases in vegetable intake as well as weight loss and other associated health benefits among overweight adults.

## Appendix

Multimedia Appendix 1 CONSORT-EHEALTH Checklist.

## Abbreviations

ANCOVA: analysis of covariance
App: mobile application
BMI: body mass index
CI: confidence interval
CONSORT: Consolidated Standards of Reporting Trials
FFQ: food frequency questionnaire
mHealth: mobile health
SD: standard deviation
SPSS: Statistical Package for the Social Sciences
US: United States
USDA: United States Department of Agriculture
